# Multiple Frames of Reference Are Used During the Selection and Planning of a Sequential Joint Action

**DOI:** 10.3389/fpsyg.2018.00542

**Published:** 2018-05-01

**Authors:** Matthew Ray, Timothy N. Welsh

**Affiliations:** ^1^Offshore Safety and Survival Centre, Marine Institute of Memorial University, St. John's, NL, Canada; ^2^Faculty of Kinesiology and Physical Education, University of Toronto, Toronto, ON, Canada

**Keywords:** joint action, shared task representations, response selection and planning, frames of reference, motor simulation, sequential joint actions

## Abstract

Co-actors need to anticipate each other's actions to successfully perform joint actions. The frames of reference (FOR) used to simulate a co-actor's action could impact what information is anticipated. We hypothesized that co-actor's would adopt their co-actor's body-centered FOR, even when they do not share the same spatial orientation, so that they could anticipate body-related aspects of their co-actor's task. Because it might be beneficial to plan joint actions based on environment and body-centered information, we hypothesized that individuals would utilize multiple FORs during response planning. To test these hypotheses, participants performed a sequential aiming task where the goal was to move a wooden dowel to one of four potential targets as quickly and accurately as possible. A cue was presented at the beginning of each trial that was either 25, 50, or 75% valid. Following the cue presentation, the first person to act (*initiator)* placed the wooden dowel, anywhere they liked, in the workspace. Then, the *finisher* performed their aiming movement from the location that the *initiator* had placed the dowel. The key dependent measure was the dowel placement of the *initiator* because it provided an index of how much the *initiator* attempted to facilitate the efficient performance of the *finisher*. The results revealed that individuals adopted an allocentric FOR (dowel placement was more biased toward cued locations as cue validity increased) and partially adopted their co-actor's body-centered FOR (dowel placement was biased toward the *finisher's* body, but not toward the co-actor's contralateral space). In conclusion, multiple FORs can be used to anticipate both body- and environment-related information of a co-actor's task. It may be difficult, however, for individuals to fully adopt their co-actor's body-centered FOR when they have differing orientations.

## Introduction

Joint actions have been defined as “any form of social interaction whereby two or more individuals coordinate their actions in space and time to bring about a change in the environment” (Sebanz et al., 2006, p. 70). Examples of joint actions that occur on a daily basis include passing a bag of groceries, helping a child put on their shoes, and navigating through a crowd of individuals. Despite the fact that joint actions appear to be performed with ease and little thought, there are numerous motor and cognitive problems that must be solved to enable successful joint actions. For example, each individual movement that contributes to the joint action originates from different people with unique bodies, abilities, thoughts, and experiences. In addition, individuals engaging in a joint action occupy different locations in space and might be oriented in a variety of ways toward other co-actors, the goal, and/or other important features of the environment. Despite these issues that need to be considered when selecting and planning joint actions, individuals are able to come together in space and time to achieve shared goals.

One way that individuals can overcome the challenges of coordinating actions in space and time is by accurately anticipating the actions of co-actors (Sebanz and Knoblich, 2009). The anticipation of another's action is thought to be enabled through a process in which each individual in the group develops shared task representations and simulates their co-actor's actions in their own motor system (Sebanz and Knoblich, 2009; Vesper et al., 2010). Shared task representations are hypothesized to contain information pertaining to the shared goal, each co-actor's task for achieving that goal, beliefs, and contextual information (Vesper et al., 2010; Pezzulo, 2011). This information from the shared task representation is hypothesized to inform the simulation processes used during the anticipation of a co-actor's action. Based on the importance of shared task representations and action simulation for anticipating a co-actor's action, it is apparent that the factors that influence what information is represented and simulated will also impact what is anticipated during joint actions.

One factor that could influence the anticipation of a co-actor's task is the spatial frames of reference that are used when representing and simulating a co-actor's action. In individual actions, spatial information used for the selection and planning of actions can be represented in different frames of reference. In egocentric frames of reference, information is coded relative to its spatial relationship to an individual's own body (e.g., eyes, hands and trunk—Colby, 1998; Klatzky, 1998; Galati et al., 2010). Allocentric frames of reference are environment-centered and information is coded based on the spatial relationship between objects or places in the environment (Colby, 1998; Klatzky, 1998; Galati et al., 2010). An additional frame of reference that needs to be considered during joint actions is the body-centered frame of reference of a co-actor. Being able to represent and simulate a co-actor's action from their body-centered frame of reference would be particularly important in scenarios where individuals are trying to predict how movement features, that can be coded to the co-actor's body, might impact the actual execution of an action (i.e., is the movement more difficult or uncomfortable based on the spatial relationship of the co-actor's body, effector and the environmental goal). Depending on the type of joint action context, critical action-related information could be coded in egocentric, allocentric and/or the body-centered frame of reference of a co-actor. Therefore, the frame of reference used when representing and simulating a co-actor's action could influence what is predicted, and hence, how effectively co-actors plan their actions to achieve the shared goal.

Pezzulo et al. (2013) have developed a shared action space framework to describe the frames of reference that might be used during joint actions. They have proposed that shared action spaces develop based on the same mechanisms that recalibrate spatial representations during tool use. Their framework is based on the notion that different frames of reference would be used in different contexts and that learning is critical in the development of these shared action spaces. For instance, they have hypothesized that goals (congruent, competitive, complementary), spatial orientation (angular disparity between co-actors), type of perspective taking [what another individual perceives (level 1 perspective) vs. how another individual experiences or would act in the world (level 2 perspective)], social factors (parent and child), and the complexity of an action all influence the frame(s) of reference adopted during joint actions.

In the simplest scenario, when co-actors share the same viewpoint and/or they only need to consider what each other can perceive, and the task requirements are low, co-actors might adopt a merged egocentric perspective—one in which each individuals' egocentric action space is combined and represented in a shared action space. However, when individuals need to consider how a co-actor will actually experience their executed action and they do not shared the same viewpoint then a more complicated scenario emerges. In this case, if there is a large angular disparity between co-actors and they have opposite spatial codes (R/L) relative to their body and the environment, then adopting the body-centered frame of reference of a co-actor would depend on complex spatial transformations to align the frames of reference. Due to the complexity of these transformations, co-actors may adopt different frames of reference to perform joint actions together. Therefore, Pezzulo et al. (2013) suggested that in those complex joint action contexts, multiple frames of reference could be adopted. By considering the proposals of Pezzulo et al. (2013), it becomes clear that there are numerous factors that can affect the frames of reference used in joint actions and, hence, the type of action-related information (environment-centered, body-centered, or other person body-centered) that could be anticipated and integrated into the selection and planning of joint actions. Although there is a growing body of literature on the complex processes that underlie joint action, the frames of reference that are used during the representation and simulation of a co-actor's action is one research topic that requires further attention.

The current literature that focuses on the frames of reference used during different types of joint actions provides evidence that individuals will co-represent a partner's response and take into account their co-actor's perspective. For example, previous research has revealed that co-actors will: adjust the height of their reaching trajectories based on the eye level of their co-actor (Quesque and Coello, 2014); laterally shift their pointing trajectories toward the person being addressed in communicative pointing (Cleret de Langavant et al., 2011); change their reach-to-grasp kinematics based on the relationship, relative position, and pronoun use of co-actors (Gianelli et al., 2013); spontaneously adopt the visuospatial perspective of a co-actor during a stimulus-response compatibility task (Freundlieb et al., 2016); perform slower pointing movements and increase end point hold time during communicative pointing movements (Oosterwijk et al., 2017); and partially adopt a co-actor's body-centered frame of reference during a shared negative priming task (Frischen et al., 2009) and when performing a mental rotation task in the presence of a co-actor (Böckler et al., 2011). Taken together, these studies show that individuals can take their co-actor's body-centered frame of reference into account when performing social motor behaviors. However, based on the framework put forth by Pezzulo et al. (2013), one could make the case that the task requirements (action demands, level of perspective taking required) were low and/or the angular disparity between co-actors was small. Therefore, the findings from these studies may not apply or scale to more complex joint action contexts. For example, when actions are used to communicate or signal information (Cleret de Langavant et al., 2011; Quesque and Coello, 2014; Oosterwijk et al., 2017) or no physical interaction is required (Böckler et al., 2011; Gianelli et al., 2013; Freundlieb et al., 2016), then individuals could have performed the task by using a level 1 visual perspective (i.e., what another person perceives). In contrast, when an individual is trying to anticipate how a co-actor will execute their action so that they can select and plan an action to help facilitate the achievement of a shared goal, then a level 2 visual perspective (i.e., how another person experiences or would act in the world) would be required.

Sequential joint actions (i.e., joint actions that involve multiple steps performed in a serial manner) are one type of joint action where co-actors could better achieve the shared goal if they could fully adopt each other's body-centered frame of reference during the anticipation of potential actions. Adopting the perspective of a co-actor is important because it would better enable individuals to plan actions that accommodate specific features of their co-actor's task, and hence, would help in achieving the shared goal. To date, findings in the sequential joint action literature demonstrate that when co-actors shared a similar spatial alignment to the environment and/or performed simple tasks (e.g., binary response alternatives), they adopted their co-actor's body-centered frame of reference. For example, individuals planned their actions to accommodate a comfortable grasping posture for their co-actor when manipulating a passed object (Gonzalez et al., 2011; Ray and Welsh, 2011; Meyer et al., 2013; Dötsch and Schubö, 2015; Constable et al., 2016; Scharoun et al., 2016). Overall, the research reviewed thus far is consistent with the framework of Pezzulo et al. (2013) and shows that when action and task requirements are low and/or co-actors have a small angular disparity between them (<90°), then individuals can represent space from their co-actor's body-centered frame of reference. However, there is currently a paucity of research that has explicitly investigated the frames of reference that are used in complex joint action tasks where co-actors have a large angular disparity between them (e.g., 180°). Therefore, it is unclear if, as suggested by Pezzulo et al. (2013), co-actor's will still attempt to adopt their co-actors body-centered frame of reference in these complex scenarios, due to the complex spatial transformations required to align body-centered frames of reference, or if they will adopt an allocentric frame of reference or multiple frames of reference.

Although not the primary purpose of their research, Ray et al. (2017) have provided some initial evidence that individuals can adopt their co-actor's body-centered frame of reference during the response selection and planning of a more complex sequential joint action task where co-actors did not share the same spatial alignment. Ray et al. (2017) sought to determine if individuals could represent and simulate the difficulty of their co-actor's actions and, hence, accommodate that difficulty during their response planning. The role of the *initiator* of the sequential joint action task was to place a dowel on a line in between two targets. The location that the *initiator* placed the dowel on the line determined the location from which the *finisher* would have to initiate their final reaching movement. Movement difficulty was manipulated based on the size of the targets (i.e., index of difficulty; Fitts, 1954) and the side of space of the target (reaching movements to contralateral space are slower and less accurate than reaching movements in ipsilateral space; Fisk and Goodale, 1985). Participants performed a joint version of the task and an individual version of the task (in which the same person was both the *initiator* and the *finisher*). The joint task was completed before and after the individual version of the task and comparisons in the performance of the *initiator* before and after the individual task allowed for the investigation of how first-hand motor experience would impact the response selection and planning of the *initiator*.

Ray et al. (2017) hypothesized that if the *initiator* represented and simulated the difficulty of the *finisher's* potential actions, then the *initiator* would bias the dowel placement toward the smaller target of the pair and toward targets in contralateral space. The results showed that the *initiator* planned their actions to accommodate the index of difficulty of the finisher's potential movements, whereas side of space only partially influenced their response planning, and only after first-hand motor experience (i.e., in the joint task that was performed after the individual task). These results could be interpreted as showing that the *initiator* represented and simulated the *finisher's* potential actions from the *finisher's* body-centered frame of reference. However, given that the side of space only partially influenced response planning, and only after first-hand motor experience, it still remains unclear if the *initiator* actually adopted their co-actor's body centered frame of reference. The other factor that makes it difficult to determine what frames of reference were used is that the co-actor's used mirror effectors (the *initiator* and *finisher* sat across from each other and the *initiator* used their right hand and the *finisher* their left hand). Therefore, side of space (contralateral/ipsilateral) was the same for both individuals, and hence, the *initiator* could have simulated the potential actions from their own egocentric frame of reference and not the finisher's body-centered frame of reference. Because of these issues, it remains unclear if individuals can adopt their co-actor's body-centered frame of reference during complex joint actions where co-actors do not share the same spatial alignment or if they will use an allocentric frame of reference or multiple frames of reference.

In terms of the use of multiple frames of reference, a clarification is required here. There is already some evidence that when action and task requirements are low and co-actors have a small angular disparity between them, then it appears as though multiple frames of reference can be adopted. For example, there is evidence that when individuals have to physically interact with the object that they will pass to their co-actor (e.g., Ray and Welsh, 2011; Meyer et al., 2013; Dötsch and Schubö, 2015), or synchronize the timing of imagined movements with a co-actor (Vesper et al., 2014), then individuals will use both their own body-centered frame of reference and their co-actor's body-centered frame of reference. In addition, there are findings in the joint attention literature that show that individuals might represent space from both their own body-centered frame of reference and their co-actor's body centered frame of reference (e.g., Frischen et al., 2009; Böckler et al., 2011). However, what has not been demonstrated thus far is whether individuals will represent their co-actor's portion of the task from multiple frames of reference (e.g., environment-centered and other person body-centered). This is an important point because depending on the joint action task demands, individuals may need to anticipate both body-centered and environment-centered features of their co-actor's action so that they can facilitate the achievement of the shared goal.

In summary, it is clear that to expand our understanding of the frames of reference that are used in joint actions research needs to be undertaken that utilizes a joint action task that: (1) has high task and action requirements, (2) has a high angular disparity between co-actors, and (3) allows individuals to facilitate their co-actor's task using either, or both, environment-centered and body-centered frames of reference. To that end, the present studies were designed to investigate the frames of reference used during a complex sequential joint action task where co-actors had a large angular disparity between them, and individuals could use information derived from multiple frames of reference to facilitate their co-actor's task. If different pieces of information can only be generated from simulations that occur from specific frames of reference, then the ability of co-actors to adopt different frames of reference is integral to co-actors reaching shared goals. For instance, allocentric frames of reference would be useful when there are multiple potential actions and the individuals need to consider the spatial relationship of those actions. Whereas, simulating actions from a co-actor's body-centered frame of reference would allow the increased difficulty of reaching movements in contralateral space (termed the “side of space” effect for this document) (Fisk and Goodale, 1985) or the preference for performing extensor over flexor movements to be anticipated (termed the proximity-to-body effect here) (Brown et al., 1948; Reed and Smith, 1961). The current studies were not designed to test the dominance of one frame of reference over another. Instead, the key questions of interest were whether individuals could adopt their co-actor's body-centered frame of reference during complex joint actions where co-actors do not share the same viewpoint and whether individuals could represent their co-actor's task from both allocentric and other person body-centered frames of reference during the response selection and planning of a sequential joint action.

## Experiment 1

To investigate the frames of reference adopted during sequential joint actions, individuals performed a sequential task, either alone or with a partner, which required them to move a wooden dowel as quickly and accurately as possible to one of four potential targets. During the joint version of the task, co-actors sat across from each other and the task was divided between the two individuals. The *initiator* was told that their co-actor (the *finisher*) would have to make their movement to the target from wherever they had placed the dowel on the board. Although the *finisher's* action is important to the task, the theoretically-relevant component of the task is the manner in which the *initiator* places the object for the *finisher*. Where the object is placed is important because the response selection and planning of the *initiator* can provide insight into the frames of reference used when representing and simulating the *finisher's* portion of the task (e.g., Ray and Welsh, 2011; Ray et al., 2017).

The targets were organized in a square and were equidistant from the center of the black board. The task was broken up into two steps. Prior to the initiation of a trial, a spatial cue (flash of an LED) indicated the potential target location for the trial with different degrees of predictability. In one block, the cue was non-predictive. Because each bock consisted of 48 trials, the target was at the cued location on 12 of the 48 trials (25% valid block). In the other blocks, the cue predicted the target location with 50% (24 of the 48 trials) or 75% (36 of the 48 trials) predictability. After receiving the cue, the *initiator* of the sequential joint action moved a wooden dowel anywhere they wished on the task environment, except for onto the targets themselves. The participant was told that the subsequent movement of the dowel onto the actual target needed to be initiated from wherever the dowel was placed. When the location of the dowel at the end of the first movement was recorded, one LED flashed to indicate the actual location of the target for that trial and the dowel had to be moved as quickly as possible onto the target location. Based on this design, several hypotheses and predictions were made.

The first hypothesis was that the *initiator* would adopt an allocentric frame of reference to integrate the cue validity information into their response planning. Based on this hypothesis, it was predicted that the dowel placement of the *initiator* would be influenced by both the cue probability and spatial location of the other potential targets. If the dowel placement was influenced by the cue validity and the spatial location of the other potential targets, then the dowel placement would be closer to the cued location as cue validity increased. In contrast, if the dowel placement was not influenced by cue validity and the other potential targets locations, then the dowel would not be placed closer to the cued location as cue validity increased or reflect the locations of the uncued target locations (i.e., it would be placed close to the center of the board regardless of the predictability or location of the cue).

The second hypothesis was that the *initiator* would adopt their co-actor's body-centered frame of reference during response planning, and therefore, would plan the action to accommodate the side of space effect (Fisk and Goodale, 1985) and the proximity-to-body effect (Brown et al., 1948; Reed and Smith, 1961). First, during the individual task, if the individuals planned their action from an egocentric perspective and anticipated the increased difficulty associated with movements in contralateral space, then their dowel placement should be closer to the targets in contralateral space in comparison to targets in ipsilateral space (e.g., Ray et al., 2017). In addition, if they adopted an egocentric frame of reference and anticipated the differences in initiating movements near and far from the body, then consistent with the proximity-to-body effect (Brown et al., 1948; Reed and Smith, 1961), the dowel placement should be biased toward their own body. During the joint task, if the *initiator* adopted their co-actors body-centered frame of reference, then the *initiator* should bias the dowel placement toward their co-actor's contralateral space (their own ipsilateral space) and toward their co-actor's body (closer to the co-actor's body and farther from their own body).

The third hypothesis was that the *initiator* would represent and integrate information based on multiple frames of reference into the selection and planning of sequential joint actions. In the present task, the cue validity should be represented in an allocentric frame of reference while the proximity-to-body and side of space effects should arise from a body-centered frame of reference. If, during the individual or joint task, the *initiator* adopted multiple frames of reference and integrated spatial information from these frames of reference into the response planning, then the dowel placement would be influenced by a combination of cue validity and one or both of the body related features (proximity-to-body, side of the space). In contrast, if during the individual or joint task, the *initiator* did not adopt multiple frames of reference during the selection and planning of the sequential actions, then the response would be based solely on cue validity or the body related factors (proximity-to-body, side of space), but not both.

### Methods

#### Participants

Twenty-two right-handed participants (mean age = 23.5, *SD* = 4; 6 males, 16 females) were recruited from the student population at the University of Toronto. One participant was unable to follow instructions and their data was not included in any of the analyses. All participants were naïve to the purpose of the study. Handedness was self-reported and all participants reported normal or corrected-to-normal vision. There were two separate experimental sessions with each lasting approximately 45–60 min each and participants were compensated $20 for their time. Written informed consent was given by all participants and this research complied with the Declaration of Helsinki and the procedures were approved by the University of Toronto Health Sciences Research Ethics Board.

#### Experimental set up and apparatus

**Figure 1:**
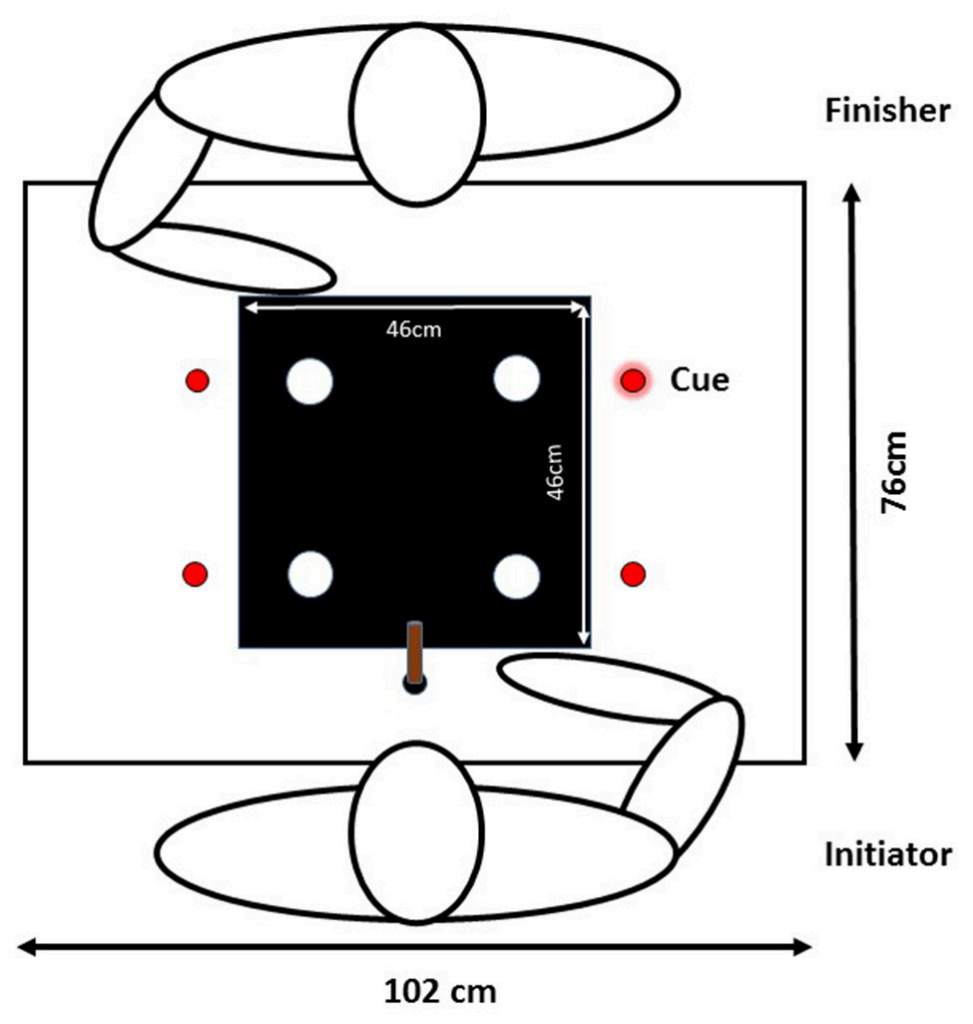
Diagram of the experimental set up for Experiment 1. Diagram depicts the presentation of a cue prior to the *initiator's* movement. The white circles are the four potential targets and the four red circles are spatial locations of the cues (i.e., light emitting diodes).

Participants sat on opposite sides of a table (see Figure 1). On top of the table, there was a foam board sheet, affixed by two C-clamps, that was 102 cm wide, 76 cm long, and 1 cm thick. On the side of the table that was closest to the participant there was a 3 cm diameter circle that served as the home position (location: 51 cm from right and left edge, and 10 cm from the bottom edge, relative to the participant). The black circle was on a piece of paper that was laminated and fixed to the foam board. In the middle of the board between both participants, there was a black square sheet of poster board (46 by 46 cm) taped to the foam board. On top of the sheet of poster board, there were four white circles (6 cm diameter) made out of poster board that functioned as target locations during the task. The four targets were equidistant to each other and were 20 cm, on a diagonal path, from the midpoint of the board.

To present cue and target location signals, four red LED lights attached to the foam board. The LED lights were at the same distance from the front and back edges of the board as the targets locations. The LEDs were 12 cm from the left and right edges of the foam board so that they were not blocked by the limbs of the individuals performing the task. The object that was moved to these targets was a wooden dowel (2.2 cm in diameter and 8 cm in height). To capture the position of the wooden dowel, an infrared light-emitting diode (IRED) from an active motion tracking system (Optotrak Certus) was attached to the center of one tip of the wooden dowel. The position of the IRED marker was recorded at 200 Hz. The tip of the wooden dowel was cut on a 45° angle so that the IRED could be easily seen by the motion tracking system. Two Dell speakers were used to present auditory signals. A Dell Optiplex 780 computer was used to run custom Matlab software and experimental output was displayed on a 19″ LCD monitor. The custom Matlab software sent signals to the speakers and the red LED lights, recorded and analyzed the dowel position, performed block and trial randomizations and organized the structure of experimental session.

#### Design and procedure

Participants performed individual and joint versions of the task on separate days. The order of the sessions was counterbalanced across participants. The experimental sessions were performed 1–2 days apart.

In the joint version of the task, the participant performed the task with a confederate (a 23 year old male who was unknown to the participants). Once the partners had been introduced and the informed consent was read and signed, verbal instructions were given. The participant and confederate were told that they were teammates and their goal was to move a wooden dowel as quickly and accurately as possible onto a target. In addition, they were told that the task was to be divided up between them and they each had very specific roles for completing the task. The participant was told that they would be the *initiator* and the confederate was told that they would be the *finisher*. The participant was told that the role assignment was random; however, the participant always performed the *initiator* role. The *initiator* was responsible for moving the wooden dowel from the home position and placing it anywhere they wanted on the black poster board sheet that contained the targets (but not on one of the actual targets). In addition, they were told that the *finisher* would have to make their movement to the target from wherever the *initiator* had placed it down and that, although the *finisher* had to move as fast as possible, there was no time constraint for the *initiator's* portion of the task.

The team was told that prior to the beginning each trial, there would be a cue that indicated the potential target location and that, depending on the block, the cue validity was either random/non-predictive (25% valid) or predictive (50 or 75% valid). The cue was signaled via a flash of light from a red LED that spatially corresponded to one of the target locations. The teammates were told that the cue validity remained constant during a block and that they would be told the cue validity at the beginning of each block. The initiator performed their portion of the task following the cue presentation. Once the initiator placed the dowel on the black square, they released the dowel and then the finisher grasped the dowel and waited for one the four red LEDs to flash and signal the actual target location.

The participants were told that there were three blocks; one block for each cue validity. The block order was counterbalanced across participants. In each block there were 48 trials. Each target location was cued 12 times per block. In the 25% cue validity block, the target appeared in the same location as the cue approximately one out of every four trials (three out of the 12 trials for that cued location). The target locations for the remaining nine trials were divided evenly between the other three target locations (three trials per location). Thus, target location was random with respect to the cue. In the 50% cue validity block, the target was at the same location as the cue on six out of 12 trials; on the remaining six trials the location of the target was divided evenly between the remaining three locations (two trials per location). For the 75% cue validity block, the target was in the same location as the cue for nine out of the 12 trials for a particular cued location. For the remaining three trials, the actual target location was divided evenly between the remaining target locations (one trial per location). The cue and target locations were chosen, according to the parameters mentioned above, via a randomization procedure using custom Matlab software.

Each trial began with the teammates sitting across from each other and the dowel in the home position in front of the *initiator*. Following the cue presentation, which occurred after a variable foreperiod (range of 100–1,000 ms), the initiator placed the dowel on the black sheet and the dowel location was recorded. The dowel position was displayed to the experimenter who verified that the recording procedure had worked. If there were any recording issues, then the experimenter was prompted to record another sample of the dowel position. Sample recordings were repeated until a valid dowel position was recorded for each trial. Once the location of the dowel was recorded, the finisher grasped the dowel and waited for the target location to be signaled. Following a variable foreperiod (range of 100–1,000 ms) that was determined by a randomization procedure in Matlab, the target location was signaled by a flash of light from one of the LEDs. The *finisher* moved the dowel as quickly and accurately as possible to the target location. Once the movement was finished, an auditory beep was presented for 50 ms to signal to the *initiator* that they could bring the dowel back to the home position for the next trial.

The trial procedure for the individual version of the task was similar to the joint version of the task except that the individual performed both the *initiator* and *finisher* roles. Therefore, the key difference was that after the cue presentation and dowel placement, the participant continued holding on to the dowel until the target location was signaled. Once the dowel position was recorded, the target location was signaled and then the dowel was moved as quickly and accurately as possible to the target.

#### Data analysis

The current experiment was designed to determine if dowel placement, following the presentation of a cue, was influenced by cue validity, the side of space effect and/or the proximity-to-body effect. The main dependent measure for this study was the distance, in millimeters, that the dowel was placed relative to the cued location. The dowel position was recorded in absolute X and Y coordinates; therefore, each data point had to be transformed to a relative distance, in the X and Y coordinates, to the cued location. Separate statistical analyses were performed on the X and Y coordinates. The analysis in the X coordinate would reveal differences in ipsilateral and contralateral space, whereas the Y coordinate analysis would reveal differences in near and far space relative to the position of the participant. The relative distance to the cued location was calculated by taking the absolute position of the dowel and subtracting the distance to the center of the cued location. The cued locations were coded relative to their position to the initiator. Because the initiator used their right hand to perform the task, the cued locations on the right side of space were coded as ipsilateral and the cued locations on the left side of space were coded as contralateral. In addition, the two cued locations on the bottom row, relative to the *initiator* (always the participant), were coded as near locations and the two cued locations closer to the finisher were coded as far locations. Because the confederate was in the opposite side of space and used their right hand all of the spatial coding is reversed; therefore, ipsilateral space is the *finisher*'s contralateral space and near space is the *finisher's* far space. The data were also coded based on the cue validity (25, 50, and 75%) and task (individual vs. joint).

### Results

#### Absolute data

**Figure 2:**
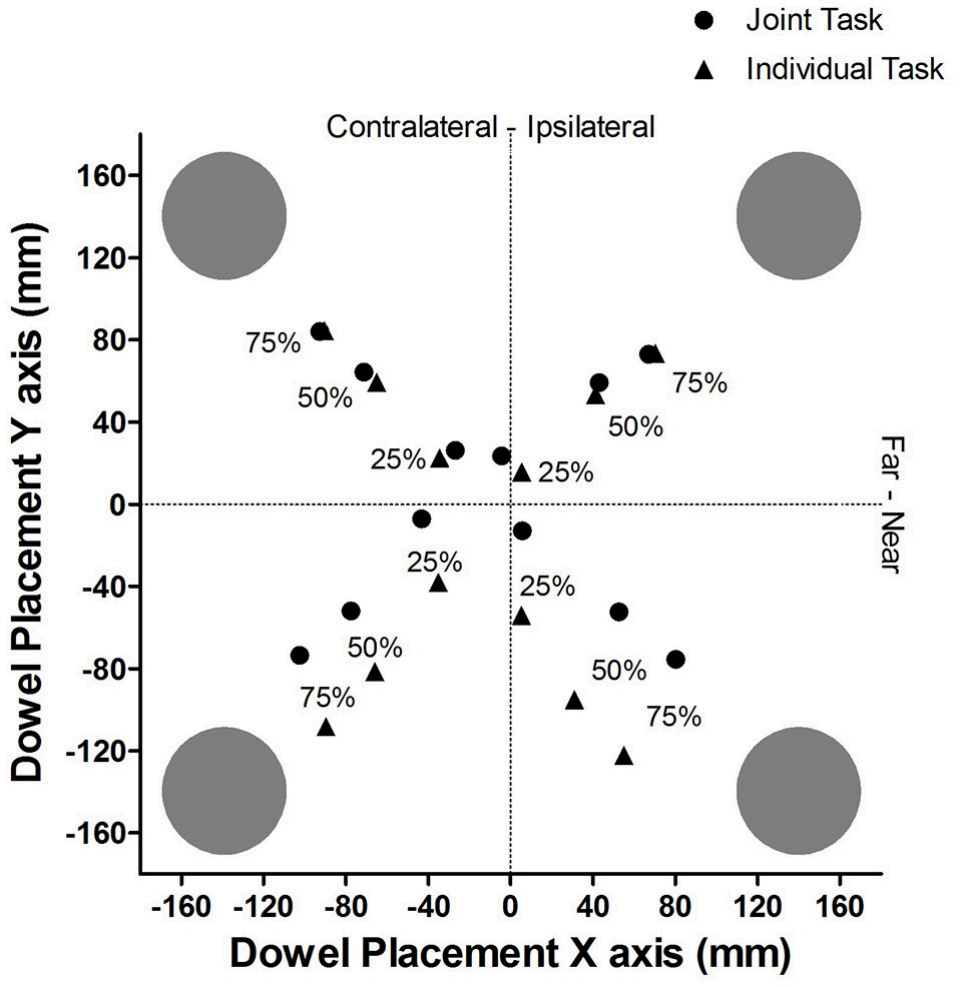
Experiment 1: Mean dowel placement in absolute coordinates for each cue validity and task condition.

Figure 2 provides a pictorial representation of the average placement of the dowel, in absolute coordinates for each cue validity and task condition. The dotted lines represent the midline for both the X axis and the Y axis.

#### Differences in the distances to cued locations along the Y axis (near to far)

The Y coordinate data was analyzed with a mixed model ANOVA with 2 (task context: Individual, Joint) x 3 (cue validity: 25, 50, 75) x 2 (side of space: Ipsilateral, Contralateral) x 2 (proximity-to-body: Near, Far) as repeated measures factors and Order (Joint task first, Individual task first) as the between-subjects factor. This analysis revealed statistically significant main effects for task context, *F*_(1, 19)_ = 7.81, *p* = 0.012, cue validity, *F*_(2, 38)_ = 46.72, *p* < 0.001, and proximity-to-body, *F*_(1, 19)_ = 4.90, *p* = 0.039. The main effects for side of space, *F*_(1, 19)_ = 0.24, *p* = 0.632 and order, *F*_(1, 19)_ = 0.690, *p* = 0.797, were not statistically significant. The full ANOVA table is presented in Appendix A.

**Figure 3:**
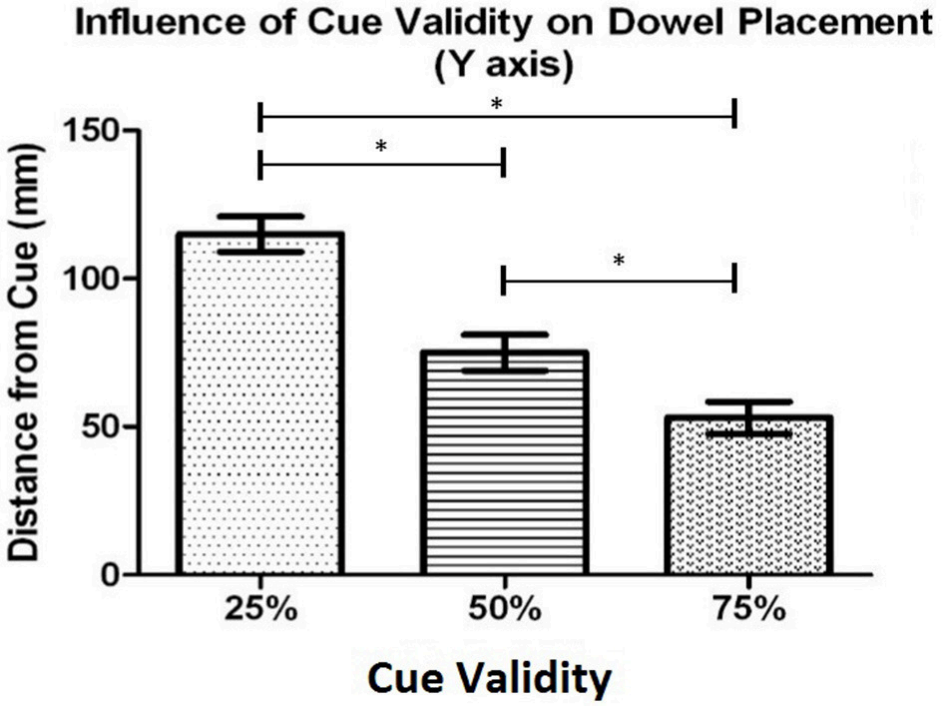
Experiment 1: Mean distance of the dowel from the cued location for each cue validity condition. Error bars represent the standard error of the mean. The ^*^ indicates a statistically significant difference.

*Post-hoc* testing of effects with 3 or more means was completed using the Bonferroni (Dunn's test) correction. The task context analysis revealed that, overall, when participants performed the individual task (*M* = 72.5 mm, *SD* = 29.8) they placed the dowel closer to the cued location in comparison to when they performed the task with a partner (*M* = 89.8 mm, *SD* = 19.1). The cue validity analysis (see Figure 3) showed that the dowel was placed significantly closer to the cued location when the cue was 75% valid (*M* = 53.3 mm, *SD* = 25.0) than when the cue was 50% valid (*M* = 75.4 mm, *SD* = 28.4), *t*_(20)_ = 4.47, *p* < 0.001, and 25% valid (*M* = 114.6 mm, *SD* = 26.7), *t*_(20)_ = 8.35, *p* < 0.001. In addition, the dowel was placed significantly closer to the 50% cue in comparison to the 25% cue, *t*_(20)_ = 5.94, *p* < 0.001. The proximity-to-body analysis revealed that the dowel was placed closer to the cued location when the cue was in Near space (*M* = 75.6 mm, *SD* = 28) in comparison to when the cue was in Far space (*M* = 86.6 mm, *SD* = 17.2). Taken together, these results show that the dowel was placed closer to the cued location, along the Y axis during the individual task. In addition, when the cued location was in the Near space, relative to the *initiator's* body, the dowel was placed closer to the cued location in comparison to when the cued location was in Far space. Lastly, this pattern of effects also shows that as cue validity increased the dowel was placed closer to the cued location.

**Figure 4:**
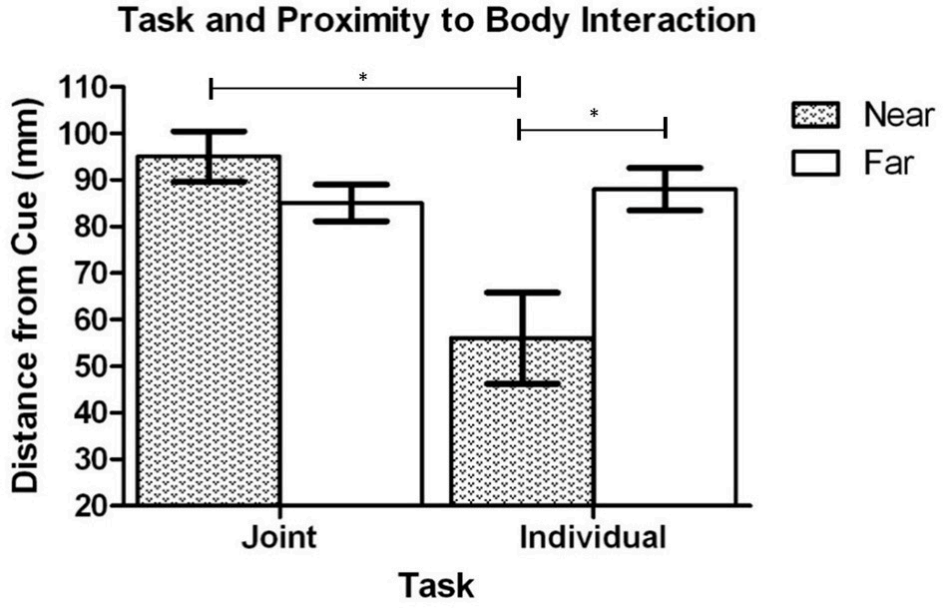
Experiment 1: Mean distance of the dowel to the cued location in Near and Far space during the Individual and Joint tasks. Error bars represent the standard error of the mean. The ^*^ indicates a statistically significant difference.

The results of the ANOVA analysis also showed that there was a significant interaction between task context and proximity-to-body, *F*_(1, 19)_ = 19.46, *p* < 0.001 (see Figure 4). *Post-hoc* analysis revealed that when the cue was presented in Near space (to the *initiator*), the dowel was placed closer to the cued location along the Y axis in the Individual task (*M* = 56.8 mm, *SD* = 45.0) in comparison to the Joint task (*M* = 94.5 mm, *SD* = 25.3), *t*_(20)_ = 3.78, *p* = 0.001. In contrast, when the cue was in Far space (relative to the *initiator*) there was no significant difference in the distance that the dowel was placed between the Individual task (*M* = 88.2 mm, *SD* = 21.4) and the Joint task (*M* = 85.1 mm, *SD* = 18.3), *t*_(20)_ = 0.82, *p* = 0.442. To determine how the dowel placement in Near and Far space varied as a function of task context additional *post-hoc* testing was performed. That analysis showed that during the Individual task the dowel was placed closer to cued locations in Near Space (*M* = 56.8 mm, *SD* = 45.0) in comparison to the Far Space (*M* = 88.2, *SD* = 21.4), *t*_(20)_ = 3.87, *p* = 0.001. In contrast, the analysis of the Joint task did not show a statistically significant difference between the Near Space (*M* = 94.5 mm, *SD* = 25.3) and Far space (*M* = 85.1, *SD* = 18.3), *t*_(20)_ = 1.97, *p* = 0.063. Overall, this pattern of effects reveals that in the individual task the placement of the dowel was clearly biased toward the *initiator's* body. In contrast, during the joint task the dowel placement was biased toward the *finisher's* body in Near space but not Far space.

Finally, the analysis also revealed an interaction between proximity to the *initiator's* body and side of space, *F*_(1, 19)_ = 22.62, *p* < 0.001. In ipsilateral space, the dowel was placed closer to the cued location when it was in Near space (*M* = 71.3 mm, *SD* = 30.7 mm) in comparison to when it was in Far space (*M* = 90.3 mm, *SD* = 17.7), *t*_(20)_ = 3.54, *p* = 0.002. There were no significant differences between Near space (*M* = 79.9 mm, *SD* = 27.2) and Far space (*M* = 83.2 mm, *SD* = 18.2), *t*_(20)_ = 0.66, *p* = 0.51, in the contralateral side of space relative to the *initiator*. This interaction reveals that the dowel was placed closer to the *initiator's* body in ipsilateral space in comparison to contralateral space. Note that the three-way interaction between task context, proximity-to-the body and side of space was not statistically significant, *F*_(1, 19)_ = 4.00, *p* = 0.060, indicating that task context did not influence the interaction between body and side of space in dowel placements.

#### Differences in the distances to cued locations along the X axis (left to right)

To determine what factors influenced dowel position placement in the X coordinate, a 2 (task context: Individual, Joint) x 3 (cue validity: 25, 50, 75) x 2 (side of space: Ipsilateral, Contralateral) x 2 (proximity-to-body: Near, Far) repeated measures mixed model ANOVA with Order (Joint task first, Individual task first) as the between-subjects factor was conducted. There were statistically significant main effects for cue validity, *F*_(2, 38)_ = 49.65, *p* < 0.001, and side of space, *F*_(1, 19)_ = 32.62, *p* < 0.001. In contrast, the main effects for proximity-to-body, *F*_(1, 19)_ = 2.17, *p* = 0.157, task, *F*_(1, 19)_ = 0.47, *p* = 0.501, and order, *F*_(1, 19)_ = 0.52, *p* = 0.481, did not reach statistical significance. The full ANOVA table is presented in Appendix B.

**Figure 5:**
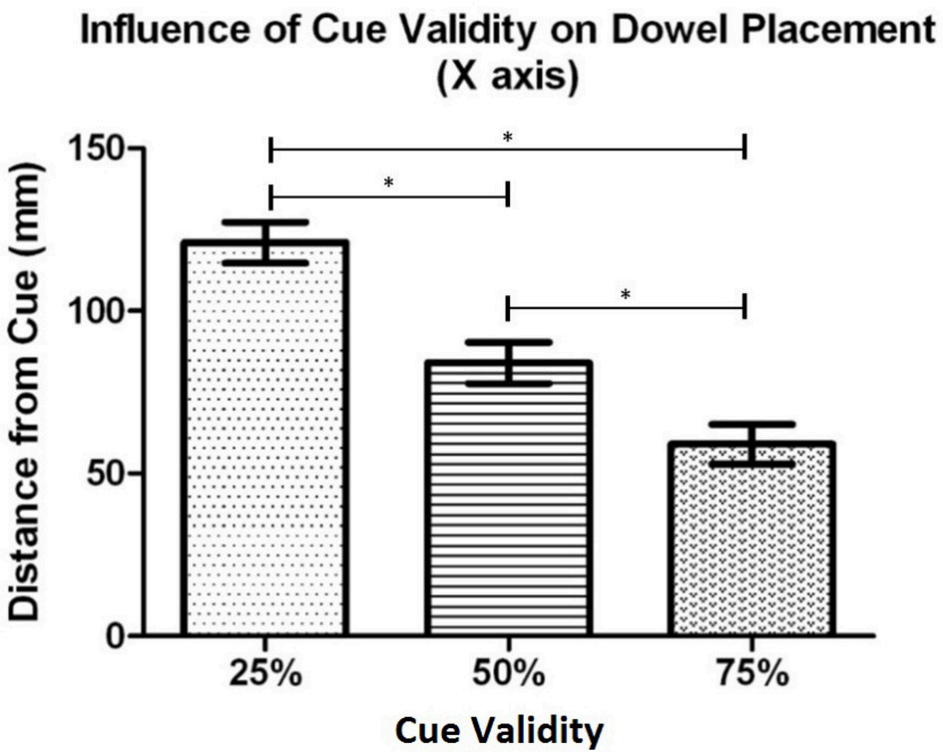
Experiment 1: The mean distance that the dowel was placed from the cued location along the X axis (left and right space) for each cue validity condition. Error bars represent the standard error of the mean. The ^*^ indicates a statistically significant difference.

*Post-hoc* testing, using Bonferroni's adjustment, was completed to determine differences in the levels of the main effects. The cue validity analysis (see Figure 5) showed that the dowel was placed significantly closer to the cued location when the cue was 75% valid (*M* = 59.0 mm, *SD* = 27.5) than when then when the cue was 50% (*M* = 84.0 mm, *SD* = 29.0), *t*_(20)_ = 5.48, *p* < 0.001, and 25% (*M* = 120.9 mm, *SD* = 29.0), *t*_(20)_ = 8.17, *p* < 0.001. In addition, the dowel was placed significantly closer to the cue when the cue was 50% valid (*M* = 84 mm, *SD* = 29.0) in comparison to the 25% valid cue (*M* = 120.9 mm, *SD* = 29.0), *t*_(20)_ = 5.34, *p* < 0.001. The side of space analysis revealed that the dowel was placed closest to the cue when the cue was in contralateral space relative to the *initiator's* body (*M* = 73.7 mm, *SD* = 23.9 mm) than when the cue was in ipsilateral space (*M* = 102.6 mm, *SD* = 27.3), *t*_(20)_ = 5.54, *p* < 0.001. Overall, these results for cue validity indicate that the dowel was placed closer to the cued location as cue validity increased. In addition, when the cued location was in contralateral space (relative to the initiator), the dowel was placed closer to the cued location than when the cued location was in ipsilateral space.

For additional results, please see Appendix C.

### Discussion

Experiment 1 was designed to test if: (1) the *initiator* of a sequential joint action adopted the *finisher's* body-centered-frame of reference, even though there was a high angular disparity between them, and (2) the *initiator* adopted multiple frames of reference during the anticipation of the *finisher's* action, and hence, planned an action that accommodated multiple action features that are represented in different frames of reference. The following sections will address these hypotheses and findings.

The first hypothesis was that an allocentric frame of reference would be adopted to utilize the cue validity information during response planning. The prediction was that the dowel would be placed, by the *initiator*, closer to the cued location as cue validity increased while simultaneously minimizing the distance to the other potential target locations. The results were congruent with the use of an allocentric frame of reference during the response selection and planning of the *initiator*. Specifically, the dowel was placed closer to the cued location as the cue validity increased and when the cue validity was low the dowel was placed in a location that was a similar distance to all the other potential actions. This finding builds on previous research by showing that when critical environment-related information which can be used to facilitate the achievement of the shared goal is available, individuals can use an allocentric frame of reference during the anticipation of a co-actor's action. The following section will explore whether individuals were able to adopt their co-actor's body-centered frame of reference.

In certain joint action contexts, if individuals adopted their co-actor's body-centered frame of reference, then they might be able to anticipate and accommodate action features that are body-based during response planning (e.g., posture). Therefore, an additional hypothesis was that the *finisher's* body-centered frame of reference would be adopted by the *initiator* when they anticipated the *finisher's* potential actions, even though there was a large angular disparity between the co-actors, and hence, body-related action features (side of space effect, proximity-to-body effect) would be integrated into the response planning of the *initiator*. There were two results that helped to elucidate whether individuals adopted their co-actor's body-centered frame of reference.

The first result, based on the Y axis (near and far from the initiator) analysis, showed that the proximity-to-body effect was modulated by both task context (Individual, Joint) and proximity to the initiators body (Near, Far). In the Near space condition (relative to the *initiator's* body), the dowel was placed closer to the cued location (along the Y axis) in the individual task in comparison to the joint task. Secondly, in the individual task the dowel was placed closer to the cued location in Near space and farther from the cue in Far space. Taken together, this pattern of effects indicates that in the individual task the dowel was biased toward the *initiator's* body in both near and far space (i.e., consistent with the proximity to body effect: Brown et al., 1948; Reed and Smith, 1961). Therefore, this finding indicates that a body-centered frame of reference was used in the individual task because the dowel was placed closer to the more difficult near targets (relative to the *initiator's* body) requiring flexor movements than to the far movements requiring extensor movements (see Augustyn and Rosenbaum, 2005; Ray et al., 2017 for evidence of similar biasing in a Fitts' Law task). In contrast, it remains unclear to what degree the *initiator* adopted the *finisher's* body-centered frame of reference because the dowel was only biased toward the *finisher's* body in Near space (far from the *finisher's* body) but not Far space (near to the finisher's body). If the *initiator* had adopted the *finisher's* body-centered-frame of reference during all of the trials, then the dowel should have been biased toward the *finisher's* body in both Near and Far space. However, the bias toward the *finisher's* body, in the *initiator's* Near space, does provide some evidence that body-related information was considered during response planning.

The second result, derived from the analysis of the X axis, showed a main effect for side of space (Ipsilateral, Contralateral), but no task context interaction. The X axis (left to right) dowel placement analysis showed that the dowel placement was biased toward the contralateral space (relative to the initiator), but not the *finisher's* contralateral space. The contralateral bias of the dowel placement in the individual task is consistent with the findings of Ray et al. (2017) and likely emerged because movements into contralateral space are less efficiently executed than those into ipsilateral space (Fisk and Goodale, 1985). Hence, the contralateral bias would help to equate the difficulty of the movements into each direction should the cue prove to be invalid (see also Ray et al., 2017). If the initiator had fully adopted their co-actors body-centered frame of reference, then there should have been a side of space by task context interaction due to the fact that contralateral space was the opposite side of space in the Individual and Joint tasks [i.e., a contralateral bias in the individual task and an ipsilateral bias (from the participants' perspective) in the joint task]. Such was not the case. Overall, the lack of a side of space effect and the presence of a partial proximity-to-body effect is congruent with the pattern of effects from the Frischen et al. (2009) study. In their study, the negative priming was strongest when the distracting stimuli was placed closest to their co-actor's hand. In contrast, the negative priming was stronger in the ipsilateral space of the observer and not their co-actor's ipsilateral space. Taken together, the pattern of effects from this experiment and the Frischen et al. (2009) study are consistent with the idea that when a task is complex and the co-actors are sitting opposite to one another, individuals might not completely adopt their co-actor's body-centered frame of reference (Pezzulo et al., 2013).

An additional purpose of this study was to investigate if the *initiator* adopted multiple frames of reference (i.e., both allocentric and other person body-centered) when they anticipated the *finisher's* potential actions and integrated that anticipated information into their response planning. In the joint task, the *initiator* appeared to select and plan their action based on information derived from an allocentric frame of reference (cue validity) and partially based on the body-centered frame of reference of the *finisher* (partial proximity-to-body effect), and hence, provides tentative support for the use of multiple frames of reference during the selection and planning of a joint action. This finding is consistent with the suggestion of Pezzulo et al. (2013) that during complex joint actions multiple frames of reference might be used simultaneously. This finding potentially goes one step further than previous work that has shown that individuals can represent a joint task from both their own egocentric frame of reference and their co-actor's body-centered frame of reference during joint tasks (e.g., Frischen et al., 2009; Böckler et al., 2011; Meyer et al., 2013; Vesper et al., 2014; Dötsch and Schubö, 2015), by showing that individuals may be able to represent the task from their own egocentric frame of reference, an allocentric frame of reference and a partially adopted body-centered frame of reference of their co-actor.

There is one potential design issue that potentially makes it difficult to interpret the different pattern of effects in Near and Far space in the Individual and Joint tasks. One reason why the dowel might not have been biased toward the *finisher's* body in Far space (their co-actors Near space) might be the size of the action space. Although the initiator could clearly reach into Far space, because they were able to move the dowel to those targets during the individual task, it may have been undesirable to make such large amplitude reaches due to the effort required. Curiously, the dowel placement was almost identical, in both the individual and joint tasks, in Far space. This finding might be an indication that the initiator was using their own body-centered frame of reference for response planning in Far space (and not fully accounting for the proximity-to-body effect), or it might indicate that the response planning was influenced by the distance required to reach into Far space. Therefore, to test between these competing hypotheses an additional experiment was performed (Experiment 2). The task was identical except that the action space was reduced by half to limit the distance that the initiator would have to reach into Far space during both initiator and finisher roles.

## Experiment 2

This experiment was designed to test if a smaller action space would influence how the *initiator* placed the dowel for the *finisher* in both Near and Far space. If the dowel placement in Far space was shaped by the reaching distance and not the partial adoption of the *finisher's* body-centered frame of reference, then now that the action space is smaller the dowel should be biased toward the *finisher's* body in both Near and Far space. In contrast, if the dowel placement is due to partially adopting the *finisher's* body-centered frame of reference, then the dowel placement would be biased toward the *finisher's* body in Near space but not in Far space.

### Methods

#### Participants

Nineteen new participants (right-handed; mean age = 20.7, *SD* = 3.18; 6 males, 13 females) were recruited from the student population at the University of Toronto. All participants were naïve to the purpose of the study. Handedness was self-reported and all participants reported normal or corrected-to-normal vision. There were two separate experimental sessions, each lasting approximately 45 min, and participants were compensated $15 for their time. Written informed consent was given by all participants and this research complied with the Declaration of Helsinki and the procedures were approved by the University of Toronto Health Sciences Research Ethics Board.

#### Experimental set up and apparatus

The experimental set up and apparatus were identical to Experiment 1 except for two features. In Experiment 1, the targets (6 cm diameter circles) were arranged in a square around the center of the black poster board and each target was 20 cm from the center of the board. In Experiment 2, each target was 10 cm from the center of the board. To maintain the same index of difficulty (Fitts, 1954) as the reaching movements in Experiment 1, the targets were reduced from 6 to 3 cm in diameter.

#### Design, procedure, and data analysis

The design, procedure, and data reduction and analysis was identical to that of Experiment 1.

### Results

#### Absolute data

**Figure 6:**
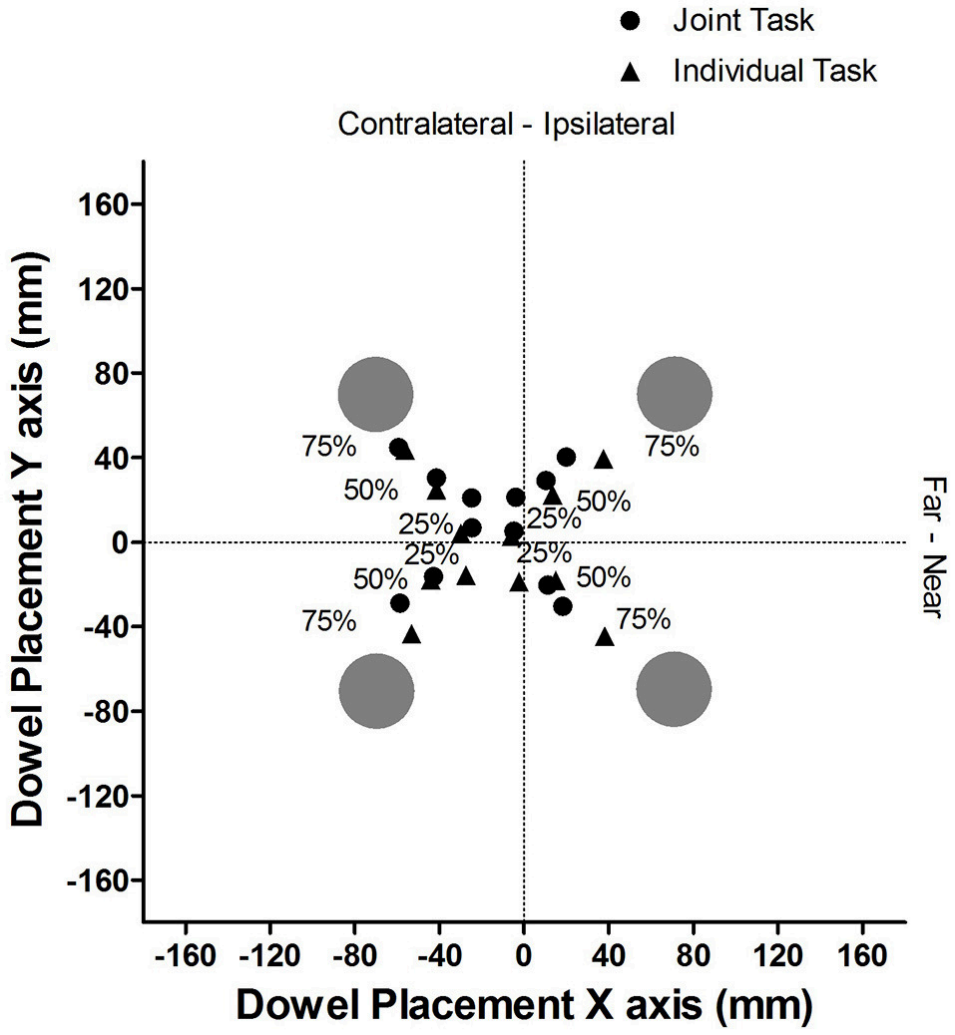
Experiment 2: Mean dowel placement in absolute coordinates for each cue validity and task condition.

Figure 6 provides a pictorial representation of the average locations of where the dowel was placed, in absolute coordinates for the different cue validity and task conditions. The dotted lines represent the midline for both the X axis and the Y axis.

#### Differences in the distances to cued locations along the Y axis (near to far)

To determine what factors influenced where the *initiators* placed the dowel the Y coordinate data was analyzed with a 2 (task context: Individual, Joint) x 3 (cue validity: 25%, 50%, 75%) x 2 (side of space: Ipsilateral, Contralateral) x 2 (proximity-to-body: Near, Far) repeated measures mixed model ANOVA with Order (joint task first, individual task first) as the between-subjects factor. There was a statistically significant main effect for cue validity, *F*_(2, 34)_ = 69.17, *p* < 0.001. However, the main effects for side of space, *F*_(1, 17)_ = 0.22, *p* = 0.645, order, *F*_(1, 17)_ = 0.71, *p* = 0.412, task context, *F*_(1, 17)_ = 0.86, *p* = 0.366, proximity-to-body, *F*_(1, 17)_ = 4.27, *p* = 0.056, did not reach statistical significance. The full ANOVA table is presented in Appendix D.

**Figure 7:**
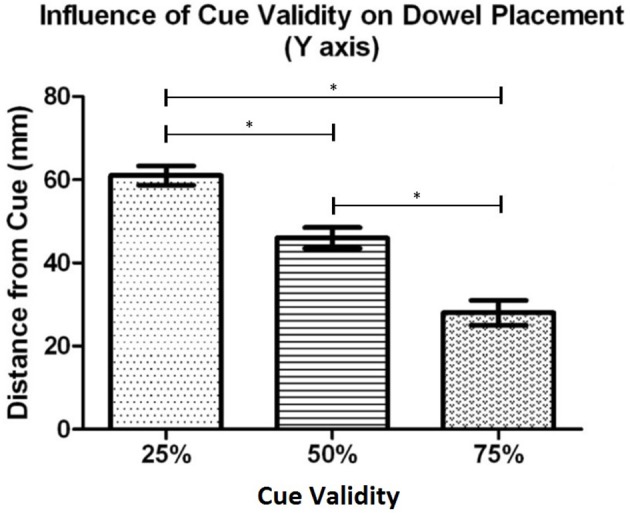
Experiment 2: Mean dowel placement relative to the cued location as a function of cue validity. Error bars represent the standard error of the mean. The ^*^ indicates a statistically significant difference.

*Post-hoc* testing, using the Bonferroni correction, on the dowel position data as a function of cue validity (see Figure 7), showed that participants placed the dowel significantly closer to the cued location when the cue was 75% valid (*M* = 28.3 mm, *SD* = 13.4) in comparison to when the cue was 50% valid (*M* = 46.4 mm, *SD* = 10.7), *t*_(18)_ = 8.47, *p* < 0.001, and 25% valid (*M* = 60.8 mm, *SD* = 10.1), *t*_(18)_ = 9.69, *p* < 0.001. In addition, the dowel was placed significantly closer the cued location when the cue was 50% valid (*M* = 46.4 mm, *SD* = 10.7) in comparison to when the cue was 25% valid (*M* = 60.8 mm, *SD* = 10.1), *t*_(18)_ = 5.29, *p* < 0.001. Similar to Experiment 1, these results demonstrate that as cue validity increased the dowel was placed closer to the cued location.

**Figure 8:**
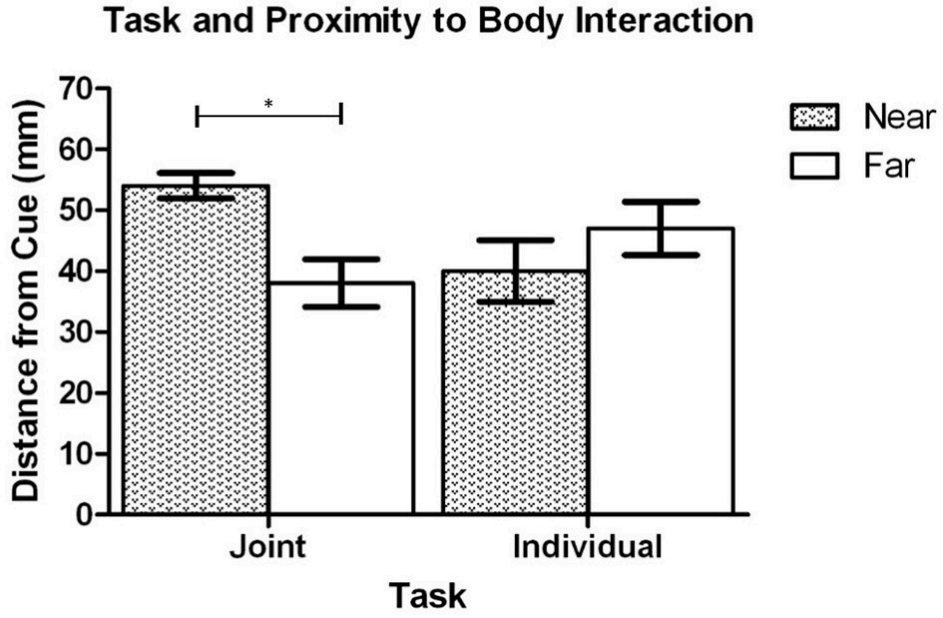
Experiment 2: Mean distance of the dowel to the cued location in Near and Far space during the Individual and Joint tasks. Error bars represent the standard error of the mean. The ^*^ indicates a statistically significant difference.

The task context and proximity to the initiators body interaction did not reach conventional levels of statistical significance, *F*_(1, 17)_ = 3.68, *p* = 0.072 (see Figure 8); however, planned comparisons were performed based on *a priori* predictions (and the results of Exp. 1). Comparisons were made between the dowel placement from the Individual and Joint tasks in both Near and Far space. In addition, separate comparisons were made between Near and Far space for the Individual task and the Joint task. Because there were four comparisons the alpha level was set at 0.013 following the Bonferroni *t* correction. The paired sample *t*-test on the dowel placement data in Near space revealed that there was not a statistically significant difference between the Individual (*M* = 40.8 mm, *SD* = 22.4) and Joint tasks (*M* = 54.2 mm, *SD* = 9.2), *t*_(18)_ = 2.35, *p* = 0.030. Similarly, the analysis of the dowel placement in Far space revealed that there was not a statistically significant difference between the Individual (*M* = 46.9 mm, *SD* = 18.8) and Joint tasks (*M* = 38.3 mm, *SD* = 17.1), *t*_(18)_ = 1.29, *p* = 0.213. The analysis of the dowel placement in the Individual task revealed that there was not a statistically significant difference between Near Space (*M* = 40.8 mm, *SD* = 22.4) and Far space (*M* = 46.9 mm, *SD* = 18.8), *t*_(18)_ = 0.80, *p* = 0.435. Lastly, the analysis of the dowel placement from the Joint Task revealed that there was a statistically significant difference with the dowel being placed closer to the cued location in Far Space (*M* = 38.27 mm, *SD* = 17.06 mm) than in Near space (*M* = 54.22 mm, *SD* = 9.15 mm), *t*_(18)_ = 3.61, *p* = 0.002. Overall, this pattern of effects demonstrates that the dowel was biased toward the *finisher's* body in the joint task, but not toward the *initiator's* body in the individual task. The proximity-to-body effect in the joint condition in Near and Far space is partially consistent with the finding from Experiment 1. However, the lack of a proximity-to-body effect in the Individual task is not fully consistent with the findings of Experiment 1.

For additional results please see Appendix E.

#### Differences in the distances to cued locations along the X axis (left to right)

To determine what factors influenced where the dowel was placed during the planning of a sequential action, the X coordinate data was analyzed with a 2 (task context: Individual, Joint) x 3 (cue validity: 25%, 50%, 75%) x 2 (side of space: Ipsilateral, Contralateral) x 2 (proximity-to-body: Near, Far) repeated measures mixed model ANOVA with Order (Individual task first, Joint task first) as the between-subjects factor. There were statistically significant main effects for cue validity, *F*_(2, 34)_ = 49.58, *p* < 0.001, and side of space, *F*_(1, 17)_ = 126.21, *p* < 0.001. The main effects for order, *F*_(1, 19)_ = 0.12, *p* = 0.732, task context, *F*_(1, 17)_ = 1.34, *p* = 0.263, and proximity-to-body, *F*_(1, 17)_ = 0.02, *p* = 0.965, did not reach statistical significance. The full ANOVA table is presented in Appendix F.

**Figure 9:**
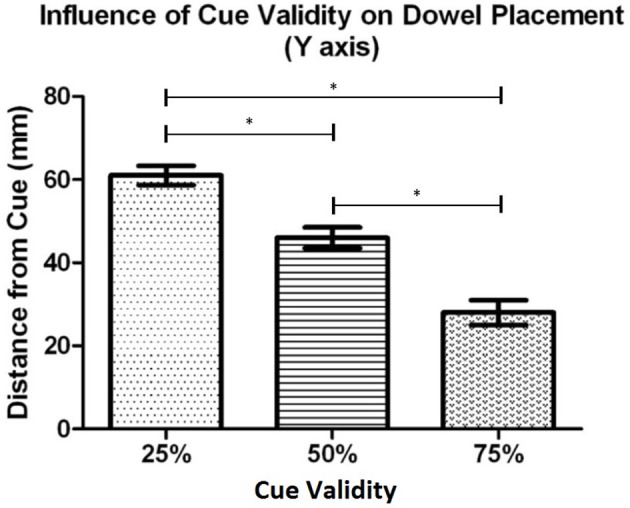
Experiment 3b: Mean distance that the dowel was placed from the cued location along the X axis (left and right space) for each cue validity condition. Error bars represent the standard error of the mean. The ^*^ indicates a statistically significant difference.

All *post-hoc* testing was completed using the Bonferroni's *t* (Dunn's test) correction based on the number of comparisons. The cue validity analysis (see Figure 9), showed that participants placed the dowel closer to the cued location when the cue was 75% valid (*M* = 27.0 mm, *SD* = 18.6) in comparison to both 50% valid (M = 42.3 mm, SD = 17.6), *t*_(18)_ = 7.09, *p* < 0.001, and 25% valid cues (*M* = 59.0 mm, *SD* = 13.7), *t*_(18)_ = 8.18, *p* < 0.001. In addition, there was a significant difference in the dowel placement between the 50% valid cue condition (*M* = 42.3 mm, *SD* = 17.6) and the 25% valid cue condition (*M* = 59.0 mm, *SD* = 13.7), *t*_(18)_ = 4.31, *p* < 0.001. These results demonstrate that as cue validity increased the dowel was placed closer to the cued location.

The side of space analysis revealed that the dowel was placed closer to the cued location when the cued location was in contralateral space (*M* = 27.78 mm, *SD* = 12.30), relative to the initiator, than in ipsilateral space (*M* = 57.7 mm, *SD* = 18.3), *t*_(18)_ = 11.37, *p* < 0.001. Again, there was no interaction between task conditions and side-of-space, *F*_(1, 17)_ = 2.34, *p* = 0.145, indicating that participants demonstrated a similar contralateral bias in dowel placement from their own perspective and did not adapt to the contralateral space perspective of the partner.

For additional results please see Appendix G.

### Discussion

The main purpose of Experiment 2 was to determine if the size of the action space influenced how the dowel was placed in Near and Far space. Therefore, the tasks from Experiment 1 were performed in a smaller action space. The key finding from Experiment 2 was that the dowel was biased toward the *finisher's* body in both Near and far Space. This finding is in contrast to Experiment 1 where the proximity-to-body effect was only seen in Near space. Therefore, the data indicates that the size of the action space did influence the dowel placement in Far space in Experiment 1. Experiment 2 also replicated the finding that cue validity influenced where the dowel was placed. Lastly, the dowel placement was only consistent with the side of space effect in the Individuals task. These findings are discussed in greater detail in the General Discussion.

## General discussion

The main purpose of the present work was to further our understanding of the frames of reference that are used to anticipate a co-actor's potential action, and hence, what environment and/or body-centered information can be integrated into the selection and planning of actions that facilitate the achievement of shared goals. To build on previous literature, we investigated if individuals could adopt their co-actors body-centered frame of reference, even though they had a large angular disparity between them and they were performing a joint task that had high action requirements. In addition, because individuals in our task could facilitate their co-actor's task using either (or both) environment and body-centered information, we went one step further than previous research and investigated if individuals would use multiple frames of reference during the anticipation of their co-actor's potential actions, so that they could accommodate both environment- and body-centered information during their response selection and planning. The following sections discuss the findings and the implications of this work in detail.

The results of both experiments provided evidence that the individuals represented their co-actors portion of the task from an allocentric frame of reference. Specifically, when the cue validity was 25% the initiator placed the dowel close to the center of the board, and therefore, a similar distance would be required to reach to any of the other four target locations. In addition, even as the cue validity increased and the dowel was placed closer to the cued location, the dowel was still placed in a location that minimized the distance to the other three targets. Because the initiator adopted an allocentric frame of reference, they were able to facilitate the *finisher's* task by reducing the impact of incorrectly anticipating the future location of a response. This finding builds on previous research that has shown that individuals will adopt their co-actor's body-centered frame of reference (e.g., Meyer et al., 2013) during the selection and planning of sequential joint actions by showing that individuals will adopt an allocentric frame of reference when they can facilitate the achievement of the shared goal based on the spatial relationship of targets, objects, and people in the shared environment.

In terms of whether the *initiator* fully adopted the *finisher's* body-centered frame of reference, during a joint action task that had demanding action requirements, the results from Experiments 1 and 2 demonstrated that the *initiator* partially adopted the body-centered frame of reference of the *finisher*. The reason for stating that the *initiator* only partially adopted the finisher's body-centered frame of reference is because the *initiator* planned actions that accommodated the distance of the dowel to the *finisher's* body (consistent with the proximity-to-body effect; Brown et al., 1948; Reed and Smith, 1961), but did not accommodate the increased difficulty of moving to targets in the *finisher's* contralateral space (which would have been consistent with the side of space effect; Fisk and Goodale, 1985; also Ray et al., 2017). If the *initiator* had fully adopted the body-centered frame of reference of the *finisher*, then the dowel placement should have been biased toward ipsilateral space (which is actually the *finisher's* contralateral space) in the Joint task. Instead, the results showed that the dowel was placed closer to the *initiator's* contralateral space (the *finisher's* ipsilateral space) in both the individual and joint contexts. To the best of our knowledge, the existing literature on the frames of reference used to anticipate a co-actor's action, during tasks that have lower action requirements and a smaller angular disparity between them, consistently shows that individuals are able to fully adopt their co-actor's body-centered frame of reference to plan actions that facilitate the comfort of their co-actor (e.g., Gonzalez et al., 2011; Ray and Welsh, 2011; Meyer et al., 2013; Dötsch and Schubö, 2015; Scharoun et al., 2016) or synchronize the timing of imagined movements (Vesper et al., 2014). Therefore, we present a novel finding by providing evidence that during more complex joint actions, where co-actors have a high angular disparity between them, individuals did not fully adopt their co-actor's body centered frame of reference.

The presence of a proximity-to-body effect, but not a side of space effect, during the Joint task, could be based on a number of factors. First, it may be that the *initiator* never anticipated the differences in difficulty of moving into different sides of space, and hence, did not plan their actions to accommodate the difficulty of moving into contralateral space. Secondly, the *initiator* may have anticipated the differences in difficulty but simply chose not to integrate this information into their response planning. Neither of these explanations seems likely given that in the individual task the response planning was influenced by the increased difficulty of moving into contralateral space (i.e., the dowel was biased toward the targets located in contralateral space). A more likely explanation is that due to the increased cognitive effort required to fully adopt the *finisher's* body centered frame of reference the *initiator* only partially adopted their co-actor's body-centered frame of reference. According to Pezzulo et al. (2013), when co-actors have a high angular disparity between them (>60–90°) and actions need to be coded based on origin dependent spatial information (e.g., laterality information), individuals need to undergo effortful spatial transformations to align opposing egocentric frames of reference. Although both the proximity-to-body effect (which is based on the spatial relationship to the body) and the side of space effect (which is based on the spatial relationship for both the hand and body) would be based on origin dependent spatial information, the coding for side of space would be more complex because both the hand and body need to be considered. For example, side of space can be coded based on laterality (i.e., left or right side of the body, left or right hand) and the side of space of the effector relative to the midline of the body (i.e., contralateral or ipsilateral), whereas, coding Near and Far space is only based on the distance to the body. Therefore, based on the pattern of effects in the present study, it appears that individuals might anticipate actions that originate from the body-centered frame of reference of a co-actor but that not all the spatial coding will be represented and simulated from their co-actor's body-centered frame of reference.

The lack of a side of space effect is somewhat congruent with a study by Ray et al. (2017) that showed that, in the first phase of the study, individuals did not plan actions to accommodate the difficulty of their co-actor's actions based on the side of space effect. However, after first-hand motor experience the dowel was biased toward targets in contralateral space. There are two variables from that study that might highlight factors that affect whether co-actors fully adopt each other's body-centered frame of reference. First, co-actors used mirror effectors (i.e., the *initiator* used the right hand while the partner in the *finisher* role used their left hand), and therefore this arrangement likely reduced (if not completely obviated) the need to fully align egocentric frames of reference. Secondly, the individual and joint task were performed in the same session and that design might have aided in the transfer of the response planning strategy from the first-hand motor experience to the joint task. In contrast, in the present study the individual and joint tasks were not performed in the same day (note also that, unlike in Ray et al., 2017 there were no statistically significant or theoretically-relevant effects of order). The interpretation that the spatial alignment (mirrored vs. opposite orientation) and learning will affect the frames of reference adopted are both consistent with the Pezzulo et al. (2013) shared space framework, which suggests that learning may be necessary to form complex shared spatial representations and that alignment is one factor that will modulate what type of frame of reference is used.

An additional purpose of the present studies was to determine if individuals represented their co-actor's task using both environment and body-centered frames of reference during complex joint actions where co-actors had a high angular disparity between them. Previous research has shown that individuals can represent a joint task from their own body-centered frame of reference and their co-actor's body-centered frame of reference (e.g., Frischen et al., 2009; Böckler et al., 2011; Ray and Welsh, 2011; Meyer et al., 2013; Vesper et al., 2014; Dötsch and Schubö, 2015; Ray et al., 2017); however, there is no evidence regarding whether or not individuals represented their co-actor's task from multiple frames of reference. In addition, because previous research had not explicitly tested if individuals could represent their co-actor's task from both environment centered and body-centered frames of reference, the tasks were not designed in such a way that individuals could facilitate their co-actor's task using either, or both, environment centered or body-centered information. Consistent with our hypothesis and the framework of Pezzulo et al. (2013), our results provide novel evidence that the *initiator* represented the *finisher's* portion of the task using multiple frames of reference during the anticipation of their potential actions. Specifically, the *initiator's* response planning was influenced by the distance to the *finisher's* body (*finisher's* body-centered frame of reference), cue validity and the spatial relationship between targets (allocentric frame of reference), and the side of space of the *initiator* (*initiators* own egocentric frame of reference). Evidence that the *initiator* adopted both the *finisher's* body-centered frame of reference and an allocentric frame of reference has already been discussed, therefore the use of an egocentric frame of reference will be discussed next.

The conclusion that the *initiator's* response planning was also based on their own egocentric frame of reference is based on the finding that the dowel was placed closer to targets in the *initiator's* contralateral space, consistent with the side of space effect, in both the individual and joint conditions. Based on this result, it would appear that action codes that concerned side of space were origin dependent on the *initiator's* body and hands. As previously mentioned, Pezzulo et al. (2013) have suggested that the most complex and effortful spatial transformations would occur when co-actors have completely opposite spatial orientations to each other (180° angular disparity) and the task requires origin dependent spatial information. Therefore, due to the difficulty and effort required to completely adopt the *finisher's* body-centered frame of reference the *initiator* may have defaulted to coding certain aspects of their task to their own egocentric frame of reference.

In addition to cognitive effort influencing the frames of reference used during joint actions, the present experiments also demonstrate that physical effort can modulate how co-actor's select and plan joint actions. Experiment 2 was conducted to investigate if the size of the action space, and hence, the amplitude of reaching movements, influenced the dowel placement in the *finisher's* near space. The results of Experiment 2 did show that when the action space was reduced the proximity-to-body-effect was observed in the *finisher's* near space. Given that the only variable that changed between Experiment 1 and 2 was the size of the action space, it seems reasonable to suggest that the lack of proximity-to-body-effect in *finisher's* near space was due to effort and not due to an inability adopt their co-actor's body-centered frame of reference. This explanation is also congruent with previous joint action research that has shown that physical effort modulates decision making in joint actions (Santamaria and Rosenbaum, 2011). The effect of effort on joint actions is clearly a topic that requires further research.

## Conclusion

The present research has revealed that during joint actions individuals will use multiple frames of reference to anticipate their co-actor's task and integrate information from those different frames of reference into their response planning. The finding that co-actors can represent their co-actor's task from multiple frames of reference is an important contribution to the joint action literature because it provides a potential mechanism for how individuals can represent both environment- and body-related factors that need to be considered during response selection and planning. In addition, our research shows that there are limitations in a person's ability to fully adopt their co-actor's body centered frame of reference. Although, previous sequential joint action research has shown that individuals fully adopted their co-actor's body-centered frame of reference and planned actions to facilitate the use of particular postures when manipulating objects (Gonzalez et al., 2011; Ray and Welsh, 2011; Meyer et al., 2013; Dötsch and Schubö, 2015; Constable et al., 2016; Scharoun et al., 2016), the present study builds on that literature by showing that when co-actors have a large angular disparity between them and they are anticipating a complex action (one that can be facilitated based on numerous response features), they might not fully adopt their co-actor's body-centered frame of reference. Instead, this study shows that when a co-actor's task can be accommodated based on a number of action features then multiple frames of reference can be used. Although the exact underlying mechanisms that support the adoption of multiple frames of reference are still unclear and beyond the scope of this paper, the present results demonstrate that when individuals are planning joint actions to accommodate aspects of a co-actor's task, they consider multiple action features based on multiple frames of reference. Future research should investigate how modulations in task complexity impact the frames of reference used during joint actions and what learning experiences are required to fully adopt a co-actor's body-centered frame of reference.

## Author contributions

MR was the primary contributor to the design, data collection and analysis, and writing while TW contributed to the design, analysis and writing.

### Conflict of interest statement

The authors declare that the research was conducted in the absence of any commercial or financial relationships that could be construed as a potential conflict of interest.
