# Supplementary material for: Multiple Frames of Reference Are Used During the Selection and Planning of a Sequential Joint Action
Source: Front Psychol. 2018 May 1;9:542. doi: 10.3389/fpsyg.2018.00542 (PMC5938383; doi:10.3389/fpsyg.2018.00542)
Supplement: Supplementary file 1 [file DataSheet1.doc]

**Appendix A**

Table 1

*Full ANOVA table for the Y Axis analysis from Experiment 1*

| Source | *Df* | *F* | *p* |
| --- | --- | --- | --- |
| Task | (1,19) | 7.81 | .012 |
| Cue Validity | (2,38) | 46.72 | .000 |
| Side of Space | (1,19) | 0.24 | .632 |
| Proximity to Body | (1,19) | 4.90 | .039 |
| Order | (1,19) | 0.69 | .797 |
| Task x Cue Validity | (2,38) | 0.22 | .808 |
| Task x Proximity to Body | (1,19) | 19.46 | .000 |
| Task x Side of Space | (1,19) | 2.69 | .117 |
| Cue Validity x Proximity to Body | (2,38) | 1.35 | .272 |
| Cue Validity x Side of Space | (2,38) | 1.46 | .246 |
| Proximity to Body x Side of Space | (1,19) | 22.62 | .000 |
| Task x Proximity to Body x Side of Space | (1,19) | 0.79 | .060 |
| Task x Cue Validity x Proximity to Body | (2,38) | 0.01 | .988 |
| Task x Cue Validity x Side of Space | (2,38) | 0.51 | .604 |
| Cue Validity x Proximity to Body x Side of Space | (2,38) | 0.87 | .426 |
| Task x Cue Validity x Proximity to Body x Side of Space | (2,38) | 0.08 | .928 |
| Task x Order | (1,19) | 0.79 | .384 |
| Cue Validity x Order | (2,38) | 0.99 | .382 |
| Proximity to Body x Order | (1,19) | 0.50 | .488 |
| Side of Space x Order | (1,19) | 0.08 | .776 |
| Task x Cue Validity x Order | (2,38) | 2.27 | .117 |
| Task x Proximity to Body x Order | (1,19) | 1.04 | .322 |
| Cue Validity x Proximity to Body x Order | (2,38) | 0.19 | .826 |
| Task x Cue Validity x Proximity to Body x Order | (2,38) | 0.97 | .390 |
| Task x Side of Space x Order | (1,19) | 1.40 | .252 |
| Cue Validity x Side of Space x Order | (2,38) | 0.39 | .681 |
| Task x Cue Validity x Side of Space x Order | (2,38) | 0.26 | .775 |
| Proximity to Body x Side of Space x Order | (1,19) | 0.61 | .443 |
| Task x Proximity to Body x Side of Space x Order | (1,19) | 0.79 | .385 |
| Cue Validity x Proximity to Body x Side of Space x Order | (2,38) | 1.47 | .243 |
| Task x Cue Validity x Proximity to Body x Side of Space x Order | (2,38) | 2.91 | .067 |

**Appendix B**

Table 2

*Full ANOVA table for the X Axis analysis from Experiment 1*

| Source | *Df* | *F* | *p* |
| --- | --- | --- | --- |
| Task | (1,19) | 0.47 | .501 |
| Cue Validity | (2,38) | 49.65 | .000 |
| Side of Space | (1,19) | 32.62 | .000 |
| Proximity to Body | (1,19) | 2.17 | .157 |
| Order | (1,19) | 0.52 | .481 |
| Task x Cue Validity | (2,38) | 1.95 | .157 |
| Task x Proximity to Body | (1,19) | 16.83 | .001 |
| Task x Side of Space | (1,19) | 0.00 | .993 |
| Cue Validity x Proximity to Body | (2,38) | 0.70 | .502 |
| Cue Validity x Side of Space | (2,38) | 1.18 | .319 |
| Proximity to Body x Side of Space | (1,19) | 2.95 | .102 |
| Task x Proximity to Body x Side of Space | (1,19) | 8.20 | .010 |
| Task x Cue Validity x Proximity to Body | (2,38) | 0.72 | .494 |
| Task x Cue Validity x Side of Space | (2,38) | 0.64 | .533 |
| Cue Validity x Proximity to Body x Side of Space | (2,38) | 0.04 | .966 |
| Task x Cue Validity x Proximity to Body x Side of Space | (2,38) | 2.44 | .101 |
| Task x Order | (1,19) | 2.08 | .165 |
| Cue Validity x Order | (2,38) | 3.03 | .060 |
| Proximity to Body x Order | (1,19) | 0.52 | .481 |
| Side of Space x Order | (1,19) | 1.80 | .196 |
| Task x Cue Validity x Order | (2,38) | 1.64 | .207 |
| Task x Proximity to Body x Order | (1,19) | 0.99 | .333 |
| Cue Validity x Proximity to Body x Order | (2,38) | 1.69 | .199 |
| Task x Cue Validity x Proximity to Body x Order | (2,38) | 2.60 | .088 |
| Task x Side of Space x Order | (1,19) | 0.91 | .353 |
| Cue Validity x Side of Space x Order | (2,38) | 0.82 | .446 |
| Task x Cue Validity x Side of Space x Order | (2,38) | 1.09 | .346 |
| Proximity to Body x Side of Space x Order | (1,19) | 2.82 | .109 |
| Task x Proximity to Body x Side of Space x Order | (1,19) | 0.17 | .688 |
| Cue Validity x Proximity to Body x Side of Space x Order | (2,38) | 0.62 | .543 |
| Task x Cue Validity x Proximity to Body x Side of Space x Order | (2,38) | 0.37 | .692 |

**Appendix C**

Post hoc testing for X axis interactions of Experiment 1

The results of the ANOVA analysis of the X axis data also showed that there was a significant interaction between task context (Individual, Joint) and proximity to the initiators body (Near vs Far space), *F*(1, 19) = 16.83, *p* = 0.001. Post hoc testing of the key conditions revealed that in Near space (to the initiator) there was no statistically significant difference in dowel placement between the Individual task (M = 79.72 mm, SD= 32.41 mm) in comparison to the Joint task (M = 92.98, SD = 27.85 mm), *t*(20) = 1.65, *p* = 0.114. In addition, in far space (relative to the initiator) there was no significant difference, in the distance that they dowel was placed relative to the cued location, between the Individual task (M = 90.58 mm, SD = 28.38 mm) and the Joint task (M = 88.67 mm, SD = 29.32 mm), *t*(20) = 0.25, *p* = 0.809.

There was also a significant three-way interaction between task context, proximity to the initiators body and side of space, *F*(1, 19) = 8.20, *p* = 0.01. Post hoc testing comparing the key conditions revealed that the dowel placement in Ipsilateral/Far space was not significantly different between the individual (M = 100.73 mm, SD = 37.63 mm) and joint tasks (M = 104.83 mm, SD = 28.97 mm), *t*(20) = 0.47, p = 0.646. In addition, the dowel placement in Contralateral/Far space was not significantly different between the individual (M = 76.60 mm, SD = 27.39 mm) and joint tasks (M = 76.33 mm, SD = 34.83 mm), *t*(20) = 0.04, *p* = 0.972. In Ipsilateral/Near space the dowel placement was not significantly different between the individual (M = 109.62 mm, SD = 36.14 mm) and joint tasks (M = 93.91 mm, SD = 34.75 mm), *t*(20) = 1.73, *p* = 0.099. Lastly, the dowel placement in Contralateral/Near space was not significantly different between the individual (M = 76.34 mm, SD = 24.11 mm) and joint tasks (M = 65.52 mm, SD = 33.42 mm), *t*(20) = 1.41, *p* = 0.173

**Appendix D**

Table 3

*Full ANOVA table for the Y Axis analysis from Experiment 2*

| Source | *Df* | *F* | *p* |
| --- | --- | --- | --- |
| Task | (1,17) | 0.86 | .366 |
| Cue Validity | (2,34) | 69.12 | .000 |
| Side of Space | (1,17) | 0.22 | .645 |
| Proximity to Body | (1,17) | 4.22 | .056 |
| Order | (1,17) | 0.71 | .412 |
| Task x Cue Validity | (2,34) | 3.56 | .039 |
| Task x Proximity to Body | (1,17) | 3.68 | .072 |
| Task x Side of Space | (1,17) | 0.92 | .352 |
| Cue Validity x Proximity to Body | (2,34) | 0.68 | .510 |
| Cue Validity x Side of Space | (2,34) | 3.84 | .031 |
| Proximity to Body x Side of Space | (1,17) | 10.92 | .004 |
| Task x Proximity to Body x Side of Space | (1,17) | 0.09 | .767 |
| Task x Cue Validity x Proximity to Body | (2,34) | 1.48 | .243 |
| Task x Cue Validity x Side of Space | (2,34) | 0.58 | .563 |
| Cue Validity x Proximity to Body x Side of Space | (2,34) | 1.61 | .214 |
| Task x Cue Validity x Proximity to Body x Side of Space | (2,34) | 1.17 | .321 |
| Task x Order | (1,17) | 13.00 | .002 |
| Cue Validity x Order | (2,34) | 1.05 | .361 |
| Proximity to Body x Order | (1,17) | 6.12 | .024 |
| Side of Space x Order | (1,17) | 4.91 | .041 |
| Task x Cue Validity x Order | (2,34) | 1.33 | .277 |
| Task x Proximity to Body x Order | (1,17) | 0.05 | .833 |
| Cue Validity x Proximity to Body x Order | (2,34) | 0.47 | .629 |
| Task x Cue Validity x Proximity to Body x Order | (2,34) | 0.63 | .538 |
| Task x Side of Space x Order | (1,17) | 0.49 | .492 |
| Cue Validity x Side of Space x Order | (2,34) | 0.43 | .657 |
| Task x Cue Validity x Side of Space x Order | (2,34) | 1.78 | .186 |
| Proximity to Body x Side of Space x Order | (1,17) | 0.23 | .636 |
| Task x Proximity to Body x Side of Space x Order | (1,17) | 3.09 | .097 |
| Cue Validity x Proximity to Body x Side of Space x Order | (2,34) | 2.51 | .096 |
| Task x Cue Validity x Proximity to Body x Side of Space x Order | (2,34) | 1.48 | .243 |

**Appendix E**

Post hoc testing for Y axis interactions of Experiment 2

The results of the analysis also showed that there were significant interactions between order and task context, *F*(1, 17) = 13.00, *p* = 0.002. Independent sample t-tests were performed to determine if there were any differences in the dowel placement in both the individual and joint task as function of order. Following the Bonferroni t correction the alpha level was 0.025, post hoc testing revealed that there was no statistically significant difference between placement in the individual task those who performed the individual task first (M = 49.8 mm, SD = 8.3 mm) and those who performed the individual task second (M = 38.5mm, SD = 12.8 mm), *t*(17) = 2.26, *p* = 0.037. In addition, there was not a statistically significant difference in placement in the joint task between those who performed the joint task first (M = 44 mm, SD = 13.6) and those who performed the joint task second (M = 48.2 mm, SD = 4 mm), *t*(17) = 0.94, *p* = 0.362.

There was also a significant interaction between proximity to the initiators body and order, *F*(1, 17) = 6.12, *p* = 0.024. Independent sample t-tests were performed to determine if there were any differences in the dowel placement in Near and Far space as function of order. Following the Bonferroni t correction the alpha level was 0.025, post hoc testing revealed that there was no statistically significant difference in the dowel placement in Near space when comparing those who performed the individual task first (M = 52.7 mm, SD = 12.8 mm) and those who performed the individual task second (M = 42.8 mm, SD = 8.7 mm), *t*(17) = 1.98, *p* = 0.064. In addition, there was not a statistically significant difference, in the dowel placement in Far space, between those who performed the joint task first (M = 40.1mm, SD = 12.7 mm) and those who performed the joint task second (M = 39.6 mm, SD = 9 mm), *t*(17) = 0.96, *p* = 0.925.

There was also a significant interaction between side of space and order, *F*(1, 17) = 4.91, *p* < 0.041. Independent sample t-tests were performed to determine if there were any differences in the dowel placement in Ipsilateral and Contralateral space as function of order. Following the Bonferroni t correction the alpha level was 0.025, post hoc testing revealed that there was no statistically significant difference in the dowel placement in Ipsilateral space when comparing those who performed the individual task first (M = 47.8 mm, SD = 10.1 mm) and those who performed the individual task second (M = 42.8 mm, SD = 7.7 mm), *t*(17) = 1.21, *p* = 0.240. In

addition, there was not a statistically significant difference in the dowel placement in Contralateral space, between those who performed the joint task first (M = 46.1 mm, SD = 11.2 mm) and those who performed the joint task second (M = 43.9 mm, SD = 8.3 mm), *t*(17) = 0.48, *p* = 0.636.

The interaction between cue validity and task context was also statistically significant, *F*(2, 34) = 3.57, *p* = 0.039. Post hoc testing compared the dowel placement in the Joint and Individual tasks at each level of cue validity (25%, 50%, 75%). Because there were three comparisons, the alpha level was set at 0.017 following the Bonferroni t correction. The post hoc testing revealed that the dowel placement, when the cue validity was 25%, was not statistically different in the individual (M = 59.1 mm, SD = 16.8 mm) and joint task (M = 62.1 mm, SD = 10.2 mm), *t*(18) = 0.68, *p* = 0.505. Similarly, there was no statistical difference between the dowel placement in the individual (M = 48.0 mm, SD = 13.7) and joint task (M = 44.7 mm, SD = 12.8), *t*(18) = 0.94, *p* = 0.359, in the 50%condition. Lastly, there was no statistical difference between the dowel placement in the individual (M = 24.4 mm, SD = 16.3 mm) and joint task (M = 32 mm, SD = 13.5 mm), *t*(18) = 2.52, *p* = 0.021, in the 75%condition.

There was also a significant interaction between cue validity and side of space, *F*(2, 34) = 3.84, *p* = 0.031. Post hoc testing compared the dowel placement in Ipsilateral and Contralateral Space at each level of cue validity. Because there were three comparisons, the alpha level was set at 0.017 following the Bonferroni t correction. The analysis of the dowel placement from the 25% cue validity condition demonstrated that there was no statistically significant difference between Ipsilateral Space (M = 60.1 mm, SD = 10.6 mm) and Contralateral space (M = 61.1 mm, SD = 9.8 mm), *t*(18) = 1.27, *p* = 0.221. Similarly, the analysis of the dowel placement from the 50% cue validity condition showed that there was no statistically significant difference between Ipsilateral Space (M = 46.2 mm, SD = 10.3 mm) and Contralateral space (M = 46.5 mm, SD = 11.8 mm), *t*(18) = 0.42, *p* = 0.683. Lastly, the analysis of the dowel placement from the 75% percent cue validity condition showed that there was no statistically significant difference between Ipsilateral Space (M = 29.2 mm, SD = 13.9 mm) and Contralateral space (M = 27.2 mm, SD = 13.4 mm), *t*(18) = 1.60, *p* = 0.126.

**Appendix F**

Table 4

*Full ANOVA table for the X Axis analysis from Experiment 2*

| Source | *Df* | *F* | *p* |
| --- | --- | --- | --- |
| Task | (1,17) | 1.34 | .263 |
| Cue Validity | (2,34) | 49.51 | .000 |
| Side of Space | (1,17) | 126.21 | .000 |
| Proximity to Body | (1,17) | 0.00 | .965 |
| Order | (1,17) | 0.12 | .732 |
| Task x Cue Validity | (2,34) | 1.36 | .271 |
| Task x Proximity to Body | (1,17) | 0.06 | .817 |
| Task x Side of Space | (1,17) | 2.34 | .145 |
| Cue Validity x Proximity to Body | (2,34) | 2.52 | .096 |
| Cue Validity x Side of Space | (2,34) | 0.47 | .632 |
| Proximity to Body x Side of Space | (1,17) | 1.06 | .318 |
| Task x Proximity to Body x Side of Space | (1,17) | 5.27 | .035 |
| Task x Cue Validity x Proximity to Body | (2,34) | 0.33 | .719 |
| Task x Cue Validity x Side of Space | (2,34) | 6.51 | .004 |
| Cue Validity x Proximity to Body x Side of Space | (2,34) | 0.98 | .633 |
| Task x Cue Validity x Proximity to Body x Side of Space | (2,34) | 0.87 | .427 |
| Task x Order | (1,17) | 1.47 | .242 |
| Cue Validity x Order | (2,34) | 2.78 | .076 |
| Proximity to Body x Order | (1,17) | 0.76 | .787 |
| Side of Space x Order | (1,17) | 0.74 | .401 |
| Task x Cue Validity x Order | (2,34) | 2.70 | .081 |
| Task x Proximity to Body x Order | (1,17) | 1.73 | .206 |
| Cue Validity x Proximity to Body x Order | (2,34) | 0.58 | .566 |
| Task x Cue Validity x Proximity to Body x Order | (2,34) | 0.03 | .974 |
| Task x Side of Space x Order | (1,17) | 0.02 | .878 |
| Cue Validity x Side of Space x Order | (2,34) | 2.22 | .124 |
| Task x Cue Validity x Side of Space x Order | (2,34) | 0.87 | .427 |
| Proximity to Body x Side of Space x Order | (1,17) | 0.01 | .928 |
| Task x Proximity to Body x Side of Space x Order | (1,17) | 0.70 | .414 |
| Cue Validity x Proximity to Body x Side of Space x Order | (2,34) | 0.46 | .633 |
| Task x Cue Validity x Proximity to Body x Side of Space x Order | (2,34) | 0.64 | .536 |

**Appendix G**

Post hoc testing of interactions for X axis data of Experiment 2

There was a three-way interaction between task context, cue validity and side of space, *F*(2, 34) = 6.51, *p* = 0.004. Post hoc tests were performed between the Individual and Joint tasks at each level of cue validity and side of space. Because there were six comparisons, the alpha level was set at 0.008 following the Bonferroni t correction. The following analyses of the dowel placement showed that during the 25% cue validity/Ipsilateral Space condition there was no significant difference between the Individual (M = 74.3 mm, SD = 17 mm) and Joint Tasks (M = 74.5 mm, SD = 26.4 mm), *t*(18) = 0.03, *p* = 0.980; during the 25% cue validity/Contralateral Space condition there was no significant difference between the Individual (M = 41.2 mm, SD = 14.3 mm) and Joint Tasks (M = 45.3 mm, SD = 19.5 mm), *t*(18) = 0.74, *p* = 0.470; during the 50% cue validity/Ipsilateral Space condition there was no significant difference between the Individual (M = 55.9 mm, SD = 25 mm) and Joint Tasks (M = 59.3 mm, SD = 23.2 mm), *t*(18) = 0.91, *p* = 0.376, during the 50% cue validity/Contralateral Space condition there was no significant difference between the Individual (M = 27.2 mm, SD = 14.4 mm) and Joint Tasks (M = 27.9 mm, SD = 17.1 mm), *t*(18) = 0.27, *p* = 0.788, during the 75% cue validity/Contralateral Space condition there was no significant difference between the Individual (M = 14.02 mm, SD = 14.32 mm) and Joint Tasks (M = 11 mm, SD = 25.7 mm), *t*(18) = 0.59, *p* = 0.566. In contrast to the findings of the previous analyses there was a significant difference in the 75% percent cue validity/Ipsilateral Space condition with the dowel being placed closer to cued location in the Individual task (M = 32.2 mm, SD = 26.7 mm) in comparison to the Joint Task (M = 50.9 mm, SD = 23.7), *t*(18) = 3.35, *p* = 0.004.

There was also a significant three-way interaction between task context, proximity to the initiator’s body and side of space, *F*(1, 17) = 5.27, *p* = 0.035. There were four comparisons, therefore the alpha level was 0.013 following the Bonferroni correction. The analysis of the dowel placement during the Near Space/Ipsilateral Space condition revealed that the dowel was placed significantly closer to cued location in the Individual Task (M = 43.17 mm, SD = 17.89) in comparison to the Joint Task (M = 61.83 mm, SD = 23.15 mm), *t*(18) = 4.36, *p* < 0.001. In contrast, the following analyses revealed that during the Near Space/Contralateral Space

condition there was no significant difference between the dowel placement in Individual (M = 28.34 mm, SD = 11.74 mm) and Joint Tasks (M = 27.98 mm, SD = 17.65 mm), *t*(18) = 0.11, *p* = 0.916, during the Far Space/Ipsilateral Space condition there was no significant difference between the Individual (M = 55.04 mm, SD = 17.78 mm) and Joint Tasks (M = 61.31 mm, SD = 22.98 mm), *t*(18) = 1.40, *p* = 0.180, and lastly, during the Far Space/Contralateral Space condition there was no significant difference between the Individual (M = 26.62 mm, SD =

10.80 mm) and Joint Tasks (M = 28.16 mm, SD = 17.37 mm), *t*(18) = 0.41, *p* = 0.685.
